# ASO Author Reflections: The Prognostic Relevance of the Proximal Resection Margin Distance is Dependent upon the Histological Subtype of Gastric Adenocarcinoma

**DOI:** 10.1245/s10434-024-15779-8

**Published:** 2024-07-10

**Authors:** Ingmar F. Rompen, Isabel Schütte, Nerma Crnovrsanin, Sabine Schiefer, Adrian T. Billeter, Georg Martin Haag, Thomas Longerich, Zoltan Czigany, Thomas Schmidt, Franck Billmann, Leila Sisic, Henrik Nienhüser

**Affiliations:** 1 Department of General, Visceral and Transplantation Surgery, Heidelberg University Hospital, Heidelberg, Germany; 2 Department of Surgery, Clarunis-University Digestive Health Care Center, St. Clara Hospital and University Hospital Basel, Basel, Switzerland; 3 Department of Medical Oncology, National Center for Tumor Diseases (NCT), Heidelberg University Hospital, Heidelberg, Germany; 4 Institute of Pathology Heidelberg, Heidelberg University Hospital, Heidelberg, Germany; 5 Department of General, Visceral, Cancer and Transplantation Surgery, University Hospital of Cologne, Cologne, Germany

## Past

For distal gastric cancer, surgical resection can be performed by either distal gastrectomy or total gastrectomy.^1^ The ability to preserve the proximal stomach is limited by the proximal tumor extent with international guidelines, such as the ESMO guidelines, which recommend a proximal margin distance (PMD) of 3 cm for intestinal-type and 8 cm for diffuse-type cancers, adding an additional safety distance.^2^ However, the integration of the PMD into guidelines is based on correlations with fewer R1 resections; but meanwhile, with reliable intraoperative frozen section, the PMD may be omitted when feasible.^3^

## Present

In the present analysis, intraoperative frozen section was found to be reliable, with less than 1% of cases having a positive proximal margin regardless.^4^ While in patients with intestinal-type tumors, oncological outcomes were not impaired when the minimum PMD was not retained, an adequate PMD was associated with improved disease-specific and overall survival in patients with cancers with noncohesive growth patterns (diffuse and mixed type).^4^ The results were comparable to those of stage II and III gastric cancer in a study by Squires et al., who, however, found a benefit of a 3 cm PMD only in stage I tumors and adjusted for diffuse histology in their multivariable analysis, but did not perform a separate analysis in that subgroup.^5^

## Future

These results may inform intraoperative decision-making in the event that the recommended PMD is not achieved. In patients with intestinal-type tumors, a completion gastrectomy may be omitted without compromising oncological outcomes. Conversely, in patients with diffuse-type cancers, associations of an inadequate PMD with worse oncological outcomes suggest the need for additional margin clearance up to a total gastrectomy. Future research should validate these findings with multi-institutional data from centers with different practices of margin handling.
